# Two decades of skeletal density decline in *Pocillopora* spp. corals in the Mexican Pacific Ocean: Insight into a tropical eastern Pacific acidification scenario?

**DOI:** 10.1371/journal.pone.0342741

**Published:** 2026-02-26

**Authors:** Andrés López-Pérez, Omar Jiménez-Gutiérrez, Héctor Reyes-Bonilla, Rafael A. Cabral-Tena, Gerardo Leyte-Morales, Francisco Medellín-Maldonado, Orión Norzagaray-López, Cecilia Chapa-Balcorta

**Affiliations:** 1 Laboratorio de Arrecifes y Biodiversidad (ARBIOLAB)/Laboratorio de Ecosistemas Costeros, Departamento de Hidrobiología, Universidad Autónoma Metropolitana-Unidad Iztapalapa, Ciudad de México, México; 2 Posgrado en Ciencias del Mar y Limnología, Universidad Nacional Autónoma de México, Ciudad de México, México; 3 Laboratorio de Sistemas Arrecifales, Departamento de Biología Marina, Universidad Autónoma de Baja California Sur, La Paz, Baja California Sur, México; 4 Departamento de Ecología Marina, Centro de Investigación Científica y de Educación Superior de Ensenada, Ensenada, Baja California, México; 5 Instituto de Recursos, Universidad del Mar, Puerto Ángel, Oaxaca, México; 6 Instituto de Investigaciones Oceanológicas, Universidad Autónoma de Baja California, Ensenada, Baja California, México; Living Oceans Foundation, TAIWAN

## Abstract

Corals demonstrate vulnerability to environmental changes, exhibiting the capacity to substantially modify coral calcification. In this study, we estimated declines in the density of *Pocillopora* coral species in the Mexican Pacific. The samples utilized in this study encompass both recently collected corals and those stored in Mexican repositories collected in the northeastern and southern Mexican Pacific regions. Density estimates indicate a 28.6% decline in coral density over the past 23 years (−0.0227 g CaCO_3_ cm^-3^ y^-1^) in the southern Mexican Pacific, while at the entrance to the Gulf of California, density has decreased by 15.4% over the past 20 years (−0.017 g CaCO_3_ cm^-3^ y^-1^). A comprehensive evaluation of environmental data reveals that the observed decline in *Pocillopora* skeletal density in Mexican Pacific reefs is concomitant with decreases in Ω_ar_ and pH, and an increase in ocean temperature on a substantial regional scale. When considered in conjunction with the previously documented reductions in coral growth of *Pocillopora* spp. skeletons in the eastern Tropical Pacific, our findings indicate a potential decline in CaCO_3_ production within the region's reef systems. The results of this study underscore the significance of generating long-term series of coral growth parameters for relevant reef-building species and the carbonate system in key and representative coastal areas, particularly those that are already challenging for coral survival and reef maintenance.

## Introduction

Coral reefs are intricate three-dimensional structures that serve as a vital foundation for maintaining elevated levels of biodiversity and providing a multitude of goods and ecosystem services [1]. The three-dimensional structure of reefs is contingent upon the accumulation of CaCO_3_ by calcifying organisms, predominantly corals [2]. However, corals are vulnerable to environmental and anthropogenic changes that have the potential to significantly alter coral calcification and, in extreme cases, threaten the maintenance of reef systems at different spatiotemporal scales [3–5]. These threats pose physiological challenges to corals, requiring modification of metabolism to adapt and acclimate. A number of parameters, including linear extension rate (cm yr^-1^), skeletal density (g cm^-3^), and calcification rate (g cm^-2^ yr^-1^), have been identified as useful in evaluating the growth rates of coral over time [3]. Consequently, growth parameters are instrumental in elucidating the impact of environmental changes on the present decline of coral populations.

Several studies have recently reported changes in coral calcification rates in response to environmental variability in various coastal regions [4,6,7]. Coral reefs are among the earliest warm-water ecosystems to be impacted by climate change. The consequences of this phenomenon are multifaceted, chiefly concerning the response of corals and other reef organisms to rising temperatures and ocean acidification [8,9]. For instance, in the eastern Tropical Pacific (ETP), coral reefs are predominantly formed by near monogeneric communities of branching *Pocillopora* type 1 (> 90% live coral cover). A coral-growth model for the equatorial ETP predicts a gradual reduction in the linear extension of *Pocillopora*, estimated at 0.9% per year, due to increased acidification [10,11]. In contrast, a decline in skeletal density has been documented in *Orbicella faveolata*, *Siderastrea siderea* and *Pseudodiploria strigosa*, while linear extension rates have remained constant. This phenomenon can be attributed to changes in the calcium carbonate saturation state, specifically the aragonite (Ω_ar_) component, within the Florida Reef Tract and the Colombian Caribbean [12,13]. Manzello et al. [14] reported a decline in skeleton density of *Porites lobata* in the southern Galapagos, while extension showed no significant trend. The authors posit that elevated nutrient concentrations in upwelled waters and/or increased heterotrophy from high water-column productivity may stimulate extension and calcification, but impair skeletal density. Concurrently, Mollica et al. [5] demonstrated through modeling that skeletal density, but not extension rate, exhibited sensitivity to ocean acidification; the model, which has been validated with data from disparate geographical regions, predicts that ocean acidification will result in a 12 ± 6% reduction in skeletal density of *Porites* by the end of the 21^st^ century [5]. In summary, although the response of corals to environmental stressors, particularly climate change, exhibits variation depending on the species and geographical region, ocean acidification and the increase in sea-surface temperature have been identified as significant stressors of coral calcification.

The majority of studies that have assessed the major drivers of variation and the temporal variability of coral calcification have focused on species that form growth bands, from which skeletal extent and density can be determined [15]. Conversely, there is a paucity of data for species with branching morphologies, including *Acropora* and *Pocillopora* species. This complicates the monitoring of changes in growth parameters for these significant reef-building species in the Caribbean and eastern Pacific, which do not form growth bands [16,17]. *Pocillopora* corals are the primary reef-building species in the ETP since the late Pleistocene epoch, and the CaCO_3_ production and reef-structural complexity of this region are closely linked to the abundance and calcification rate of these corals [17,18]. Consequently, any reduction in *Pocillopora* corals, whether attributable to natural or human-induced perturbations or calcification, could have an immediate or long-term detrimental impact on the functionality, stability, and biodiversity of the coral ecosystems in question [19]. The scarcity of data concerning the growth parameters of coral species relevant to reef system functionality is particularly disconcerting in the ETP, a region that is known to possess the most severe natural acidification conditions for reef development in the world [20,21]. The region’s environmental conditions are conducive to minimal reef accretion due to the interaction of upwelled subsurface water with low pH (7.8 as the lower threshold) and a near-unity subsaturation state of aragonite in seawater. It is anticipated that these conditions will become even more marginal for accretion in future ocean acidification scenarios [11,21–24].

This study presents empirical data on the decline in the density of *Pocillopora* type-1 skeletons over the last two decades in the Mexican Pacific. The findings are then contextualized within the progressive acidification scenario of the eastern Pacific and the potential risk to the persistence and functioning of reef systems in the region.

## Materials and methods

### Study area

**Fig 1:**
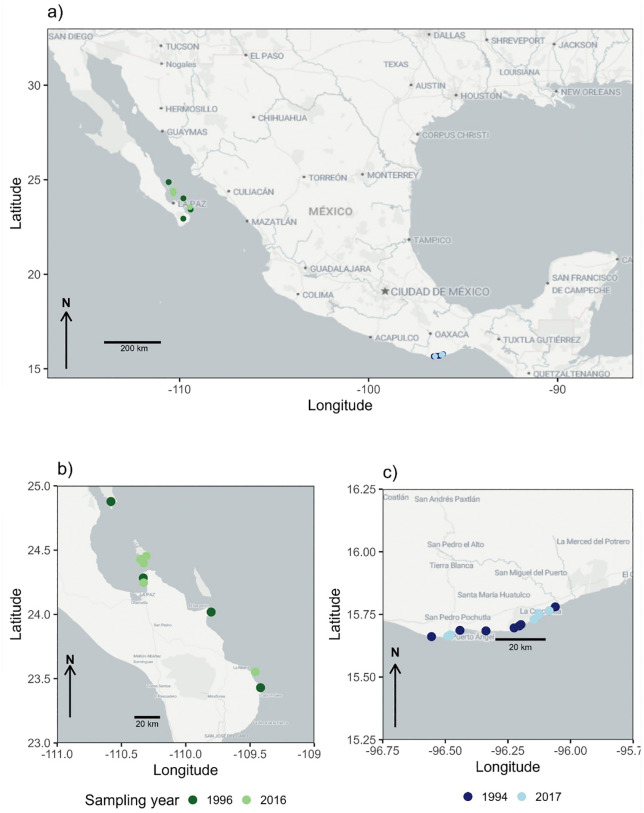
Study area and sampling sites in the Mexican Pacific (a). **(b)** Green = entrance of the Gulf of California: Isla San José, San Gabriel, Swami, Bonanza, Pichilingue, Calerita, Punta Arenas, Punta Perico, Bahía Chileno, Cabo Pulmo. **(c)** Blue = coast of Oaxaca: Mazunte, Panteón, Estacahuite, La Tijera, Salchi, Riscalillo, La Prima, La India, Maguey, La Entrega, Dársena, Isla Montosa, Guerrilla.

Sampling was conducted at multiple locations in the eastern tropical Pacific, with a particular focus on the northwestern region (entrance of the Gulf of California) and the southern region (coast of Oaxaca) of the Mexican Pacific (Fig 1). A distinguishing characteristic of both regions is their elevated degree of oceanographic dynamism, which is influenced by a variety of seasonal processes. At the entrance to the Gulf of California, anticyclonic eddies with cold-water filaments of approximately 22°C are generated by the collision of the California Current with the continent. This collision subsequently leads to the former's expansion, with a portion of it entering the Gulf [25,26]. The Gulf of California Water, a water mass of local origin, exhibits the highest salinity values in the area (≥ 34.9) and demonstrates a notable variability in temperature (> 12°C annual range). In contrast, less saline (< 34.9) and comparatively warmer Tropical Surface Water, which originates from the Pacific Ocean, enters the system [25].

The limited reports from the Gulf of California region pertaining to carbon chemistry, encompassing dissolved inorganic carbon (DIC) concentration, Ω_ar_, and pH, suggests a dependence of conditions on the predominant water masses at the seasonal scale. From spring to fall, tropical surface water is predominant, characterized by DIC values of 2008 ± 48 µmol kg^-1^, Ω_ar_ = 3.2 ± 0.3 units, and pH = 8.01 ± 0.03. In contrast, during the winter months, the Gulf of California water predominates, characterized by higher DIC values (2079 ± 27 µmol kg^-1^), the lowest Ω_ar_ (2.8 ± 0.2 units), and a pH of 7.93 ± 0.02. The mean pH, when both seasons are considered is 7.99 ± 0.05, with a range of 0.14 units (7.92–8.06) [26–29].

The coral communities situated along the coast of Oaxaca are shaped by the variable seasonal conditions that prevail in this region. Intense coastal upwelling, promoted by seasonal strong winds known as “Tehuanos”, is observed in the western region of the Gulf of Tehuantepec (the same mechanism is observed in Costa Rica [20,30]). This phenomenon occurs during the spring months of March and April. The mechanism in question gives rise to the vertical transportation of subsurface water, which is characterized by elevated concentrations of DIC (~ 2200 µmol kg^-1^) that extend along the coastline. The upwelled subsurface water is distinguished by its relatively low pH (~7.7) and Ω_ar_ values (~1.4 units). It is subsequently mixed with surface water by mesoscale structures, such as dipole eddies interacting with coastal waters [31]. Throughout the remaining months of the year, the water column situated above 100 m in depth is composed of tropical surface water, with pH values ranging from 8.2 to 8.3 and Ω_ar_ values from 2 to 2.4 [32].

### Coral sampling

The experimental design was devised to ascertain trends in skeletal density of *Pocillopora* colonies collected during two distinct periods at both localities: (1) 1996 and 2016 at the entrance of the Gulf of California and (2) 1994 and 2017 at the coast of Oaxaca (Fig 1). The experimental design was selected because *Pocillopora* do not form annual bands as massive corals do. This characteristic complicates the sclerochological analysis of the genus. In 2016, 33 fragments of *Pocillopora* spp. colonies were collected at five locations in the Gulf of California, while 34 fragments were obtained from colonies collected in 1996 at seven localities in the same area (Fig 1a–1b). Presently, these fragments are housed in the coral collection of the Natural History Museum of the Autonomous University of Baja California Sur. Conversely, in 2017, 38 coral fragments were collected at six localities on the coast of Oaxaca, while 38 fragments were obtained from colonies collected during 1994 at ten localities in the same area, which are deposited in the reference coral collection of the Universidad del Mar (Fig 1a, 1c). Information regarding the morphotypes sampled per locality, area, and time can be consulted in supplementary material (S1 Table).

In consideration of the sampled times, the studied periods were deemed to be within the ENSO-neutral variability (https://psl.noaa.gov/data/timeseries). According to the National Oceanic and Atmospheric Administration (NOAA) (https://psl.noaa.gov/data/timeseries), the estimated levels of marine heat waves were low (<1 DHW) for each of the years considered in the study (https://coralreefwatch.noaa.gov/product/5km/index_5km_dhw.php). Therefore, it is unlikely that El Niño Southern Oscillation (ENSO)-related heat stress significantly impacted calcification during the study's time frame.

Samples were collected from colonies growing at depths ranging from 1 to 9 meters. In all cases, the fragments were obtained from 2 centimeters below the tip of the branch zone. This criterion was adopted for the purpose of obtaining density estimations from coral sections, thereby circumventing the initial rapid growth reported from colonies’ tips [33]. To remove the coral tissue remnants, the fragments were immersed in a 5% sodium hypochlorite solution for a period of 24 hours [34].

### Density estimations

Due to the paucity of long-term series with which to ascertain changes in skeletal density of branching corals, we sought to obtain density data from skeletons deposited in biological reference collections. The determination of density by means of the simple ratio of weight to volume (weight/volume) of the skeleton plus the volume of the “air” represents a low-tech, robust method that allows for the accurate and reproducible determination of the density of highly porous objects, such as corals, by water freezing. Cubes measuring approximately 1 cubic centimeter were obtained from each fragment using a high-speed diamond saw (SYJ-40-LD). The density of the cubes was estimated by the freezing method, following the procedure described by Carricart-Ganivet et al. [34]. Equation (1) considers the weight of the dry fragment (*W*_*d*_), the density of aragonite (*d*_*ar*_, 2.93 g cm^-3^), the weight of the frozen fragment (*W*_*f*_), and the density of ice (*d*_*ice*_, 0.915 g cm^-3^). Skeletal density of the fragment was determined using the following formula (*d*_*b*_):


$$d_{b}=W_{d}/(W_{d}/d_{ar})+(W_{f}-W_{d})/d_{ice}$$ 1

The data presented herein constitutes a conservative estimate of the density of the branches of the colonies, excluding the thickening and widening of the branch tips.

### Environmental setting

The objective of this study is to ascertain whether changes through time in the skeletal density have occurred among select coral species within the designated study areas. Additionally, the investigation seeks to ascertain whether these decrements may be associated with changes in environmental conditions, particularly with variables associated with ocean acidification (OA) over the past two decades. Both study areas exhibit a dynamic oceanography, characterized by numerous processes occurring at varying temporal and spatial scales. These include seasonal vertical and horizontal advection of water masses, precipitation, and interannual phenomena such as El Niño Southern Oscillation (ENSO) events, to name a few.

Time series records of OA indicators (i.e., pH, DIC, saturation state regarding aragonite [Ω_ar_]) are scarce for both study areas, a common situation for many oceanic and coastal regions of the Mexican Pacific. The scenario is rendered more complex by the fact that the available data correspond to relatively short and discontinuous time periods. Moreover, the data from multiple sources exhibited considerable heterogeneity, attributable to various factors. These factors include the methodologies employed for calculation, the established best practices for data analysis, the spatial and temporal resolutions of monitoring, and additional elements contributing to the natural variability observed in both regions. The distribution of the data in an irregular pattern hinders the identification of long-term trends. A strategy employed to study long-term biogeochemical processes (i.e., OA) in areas with limited data is to utilize modeled data to complement observed data.

In this study, we have calculated the pHT (total scale pH) and the Ω_ar_ from atmospheric pCO_2_ (pCO_2atm_) and modeled total alkalinity (TA). Notwithstanding the potential oversimplification of the carbonate-system dynamics that may result from this approach, its potential application for the purposes of this study was nevertheless evaluated. To achieve this objective, monthly-averaged environmental variables (i.e., temperature, °C; silicate and phosphate concentration, μM; salinity) at annual scale for each studied site were obtained for 1° x 1° quadrats from the World Ocean Atlas 2018 (WOA18, https://www.ncei.noaa.gov/products/world-ocean-atlas). The TA (µmol kg^-1^) was calculated using the equations developed by Lee et al. [35] for subtropical (Gulf of California entrance) and tropical (coast of Oaxaca) areas (± 20 µmol kg^-1^). The calculation of Ω_ar_ and pHT was performed using the software CO2sys [36], with salinity, temperature, depth, phosphate, silicate, TA, and pCO_2atm_ utilized as the dependent variables. The pCO_2atm_ data were obtained from the CO_2_ program of the Scripps Institution of Oceanography for the designated study periods (https://scrippsco2.ucsd.edu/data/atmospheric_co2/ljo.html; considering a nominal error of 20 µatm). The pHT and Ω_ar_ estimations presented an uncertainty of 0.01–0.03 and 0.08–0.14 units, respectively (20 µmol kg^-1^ for DIC and 0.1 °C data).

To verify if calculated pHT fall within the range of measured data variability, carbon chemistry data were obtained from different sources (S2 Fig A–D). To contrast pHT and Ω_ar_ values between the different databases (S2 Fig A–D), the same carbonate dissociation constants were utilized [37]. In instances where TA data were not available (e.g., SOCAT database [38]), the necessary estimates were derived using the methodology outlined by Lee et al. [35]. This exercise facilitated the validation of the estimated data, which were determined to align with the range of observed natural variability. This finding suggests that the estimated data may be representative of the prevailing conditions in each region.

Furthermore, to ascertain the long-term shifts in pH within the two study areas, pHT and Ω_ar_ were derived from the outputs of the Community Earth System Model v2, which incorporates historical DIC, TA, temperature, and salinity data as reported by Wieners et al. [39]. The selection of this model is based on its demonstrated capacity to replicate the observed seasonal patterns and magnitudes of temperature and salinity. Despite its proclivity to underestimate average pH and smooth natural variability, this model enabled the estimation of trends in Ω_ar_ and pHT.

### Data analysis

To assess changes in coral skeletal density between areas, times, localities, and species, the present study employed a four-way nested (area (time (locality (species))) unbalanced type III permutation-based analysis of variance (PERMANOVA). The PERMANOVA was conducted on a Euclidean distance matrix with 10,000 permutations, in accordance with the criteria set forth by Anderson et al. [40].

In addition, an analysis of covariance (ANCOVA) was employed to ascertain whether the trajectories of skeletal density (i.e., the rates of change over time) vary between regions (i.e., the Gulf of California entrance versus the coast of Oaxaca). This approach enables testing the hypothesis that the slopes of each linear regression in each area are different. In this analysis, area was utilized as a categorical variable, year as a covariate, and skeletal density as the response variable. In essence, an ANCOVA was employed to investigate a significant interaction between the categorical variable (area) and the covariate (time). Prior to conducting the aforementioned analyses, the assumptions of normality and homoscedasticity were evaluated via the Kolmogorov-Smirnov and Bartlett tests, respectively.

Due to the restricted access to *Pocillopora* spp. museum samples for density determinations and the inclusion of only two time periods, the analysis of environmental variables was conducted using principal component analysis. The ordination encompassed vectors for each environmental variable, with the length and direction of the vector serving as proxies for the relative importance of each variable in the ordination.

The decrements in skeletal density undergone by *Pocillopora* spp. within the ETP over time were examined through the application of simple linear regression analysis. The coral density data utilized in the analysis corresponded to the values determined in this contribution and to the determinations published by [11,41–43].

All statistical analyses were performed at α = 0.05. Analyses were performed using Past v4.09 [44] and PRIMER & PERMANOVA+ 6.1 [40].

## Results

The findings indicated that the sole dimension of sampling across which there were statistically significant disparities in density was between time periods (Pseudo-F_(2,81)_= 15.83, p = 0.0001). The analysis revealed no statistically significant differences between areas (Pseudo-F_(1,81)_= 2.27, p = 0.27), between sites nested within time and area (Pseudo-F_(24,81)_= 1.50, p = 0.14), or between species nested within site, time and area, were observed (Pseudo-F_(28,81)_= 1.06, p = 0.4) (S4–S6 Fig).

In general, corals of the genus *Pocillopora* exhibited higher skeletal density, with an average of approximately 30% more calcium carbonate per unit volume, at coral reefs located at the entrance of the Gulf of California (2.04 ± 0.34 g cm^-3^ CaCO_3_) compared to coral reefs of Oaxaca (1.55 ± 0.39 g cm^-3^ CaCO_3_) (S5 Fig and Table 1). In 1996, the Gulf of California entrance site known as Punta Perico exhibited the highest average density value (2.23 ± 0.09 g CaCO_3_ cm^-3^), while the lowest average value was recorded at Isla San José (1.98 ± 0.04 g CaCO_3_ cm^-3^). In contrast, the mean skeletal density values for all sites along the coast of Oaxaca in 1994 were lower than those documented at the Gulf of California entrance in 1996, with the exception of Riscalillo (2.00 ± 0.14 g CaCO_3_ cm^-3^). The lowest recorded value for the coast of Oaxaca was observed at La Tijera (1.62 ± 0.76 g CaCO_3_ cm^-3^).

**Table 1:** Average environmental variability and skeletal density (± SD) of *Pocillopora* species across regions and time in the Mexican Pacific. For details on the methodology employed to obtain the environmental values, please refer to the text. SST = °C, Ω_ar_ = aragonite, skeletal density = g CaCO_3_ cm^-3^. *Significant differences (p < 0.05).

	Gulf of California entrance		Coast of Oaxaca	
	1996	2016	1994	2017
SST	24.66 (±2.70)	26.97 (±2.40)*	28.50 (±1.20)	28.97 (±1.34)
Surface pH	7.98 (±0.03)	7.95 (±0.03)*	7.99 (±0.01)	7.96 (±0.01)*
Ω_ar_	3.27 (±0.10)	3.17 (±0.08)*	3.16 (±0.12)	3.07 (±0.14)
*Skeletal density*				
*Pocillopora capitata*	2.40 (±0.10)	1.65 (±0.36)	1.85 (±0.20)	----
*Pocillopora damicornis*	2.19 (±0.21)	1.89 (±0.37)	1.91 (±0.24)	1.26 (±0.38)
*Pocillopora elegans*	----	1.94	1.13	----
*Pocillopora grandis*	----	1.94 (±0.12)	1.83 (±0.25)	1.28 (±0.08)
*Pocillopora meandrina*	2.16 (±0.27)	----	1.68 (±0.01)	1.67 (±0.03)
*Pocillopora verrucosa*	2.19 (±0.23)	2.07 (±0.34)	1.70(±0.39)	1.28 (±0.33)

A decline in skeletal density over time was observed in both areas. The density exhibited a higher value in 1994 (1.813 ± 0.08 g cm^-3^ CaCO_3_) compared to 2017 (1.295 ± 0.1 g cm^-3^ CaCO_3_) in the coastal region of Oaxaca. A similar trend was noted in the skeletal density, which exhibited a higher value in 1996 (2.21 ± 0.21 g cm^-3^ CaCO_3_) compared to 2016 (1.87 ± 0.37 g cm^-3^) in the Gulf of California entrance (S5 Fig). The aforementioned evidence indicates that there has been a notable reduction in the density of *Pocillopora* spp. skeletons. The decline in density was 28.6% over the past 23 years in Oaxaca, with a rate of decrease of approximately 0.022 g CaCO_3_ cm^-3^ in calcification per year. In contrast, a decrease of 15.4% in density was observed over the last 20 years in the Gulf of California entrance, with a rate of decline of about 0.017 g CaCO_3_ cm^-3^ in calcification per year. An analysis of covariance (ANCOVA) was employed to assess the impact of area and the continuous covariate (year) on skeletal density, incorporating the interaction between year and area to compare slopes. The findings indicated that the effect of year was significant (F_(1,133)_=70.62, p < 0.001), indicating that skeletal density undergoes a substantial decline over time. In addition, the effect of area proved to be significant (F_(1,133)_=104.27, p < 0.001), thereby indicating disparities in mean skeletal density across distinct areas. Conversely, there was no interactive effect of year and area (F_(1,133)_=2.58, p = 0.111), indicating that the rate of change in density over time is statistically comparable across regions. In the fitted model, the slope for the Gulf of California was determined to be −0.01512 g CaCO_3_ cm^-3^ y^-1^ (p = 0.0001), while for the Oaxaca area was found to be −0.0227 g CaCO_3_ cm^-3^ y^-1^ (p = 0.0001). In summary, density has exhibited a negative trend over the years, a pattern that is consistent across different regions.

Concurrently, the pCO2atm and TA models, as well as the CESM2 model, indicate a modest decline in pHT and Ω_ar_ for both regions (Table 1). This decline is evident in the data from 1995 to 2014, with a decrease of −0.001 units per year for pHT and −0.005 units per year for Ω_ar_ (S3 Fig). However, the magnitude of this decline appears to be slightly more pronounced in the Oaxaca coastline compared to the Gulf of California entrance.

**Fig 2:**
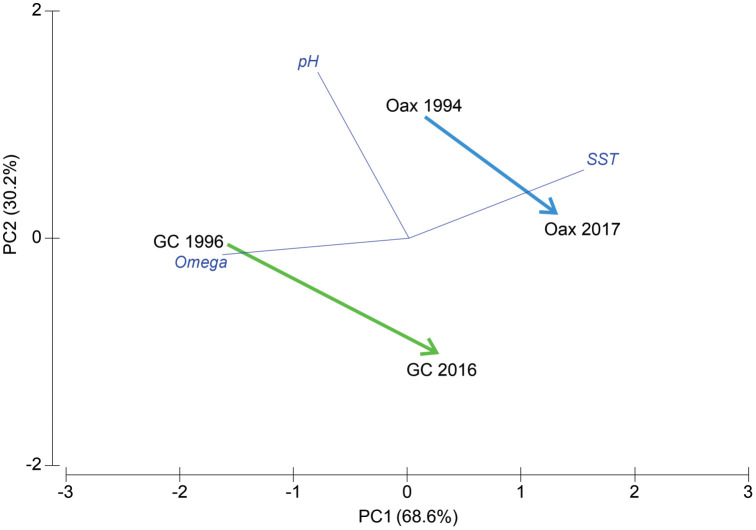
Principal component analysis (PCA) of areas and times based on environmental variables. For further details, please refer to the text. GC denotes the Gulf of California entrance, while Oax represents the coast of Oaxaca. The green color indicates the environmental change between 1996 and 2016 within the Gulf of California entrance, while the blue color represents the environmental change between 1994 and 2017 within the coast of Oaxaca. The dark blue color denotes the environmental vectors (r = 0.8), which include surface pH, surface Ω_ar_, and SST. Spatial Euclidean distance: Gulf of California entrance = 2.51, coast of Oaxaca = 1.8.

The principal component analysis explained 99.3% of the environmental variation between areas and times (Fig 2, Table 1). Along PC1 (77.8% variance explained), the upper left part of the ordination biplot displays the environmental centroids for areas and times sampled in 1994 and 1996, while the lower right part of the ordination shows the environmental centroids of areas and times sampled in 2016 and 2017. As indicated by the aforementioned evidence, the environmental conditions have undergone modifications, particularly evident among samples obtained at different times within a given area. The analysis indicated that the most influential variable in the ordination was Ω_ar_ (r = 0.68 with PC1), and in accordance with the vectors’ angle, this indicates an inverse response, exhibiting lower values towards 2016 and 2017. Conversely, temperature exhibited a positive correlation with PC1 (r = −0.64 with PC1), indicating that temperature has increased in the areas towards 2016/2017. Finally, pH demonstrates a robust positive correlation (r = 0.92) with PC2. Consequently, the areas have experienced a decrease in Ω_ar_ and pH and an increase in ocean temperature (Fig 2, Table 1). However, according to the spatial Euclidean distance, the environmental change was more pronounced at the Gulf of California entrance (2.51) than along the coast of Oaxaca (1.80).

**Fig 3:**
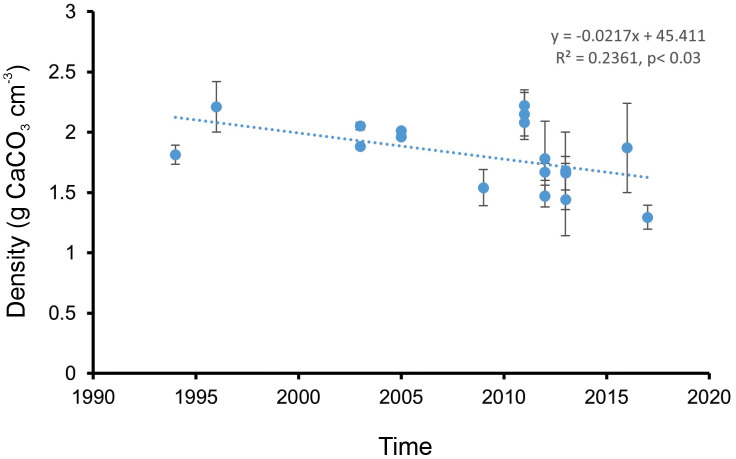
Linear trend in skeletal density in *Pocillopora* species in the eastern tropical Pacific Ocean over time. The data points represent the density values for each time period and area (combining both localities’ data), while the error bars indicate the standard deviation of density. The dotted line represents the linear regression model.

Finally, the analysis of changes in skeletal density of *Pocillopora* spp. indicates a notable decline between 1994 and 2017 in the ETP (R2 = 0.23, n = 21, p < 0.03; y = −0.0217x + 45.411) at a rate of −0.0217 g CaCO_3_ cm^-3^ per year (Fig 3).

## Discussion

The estimated density values for the various *Pocillopora* species in this study, regardless of the area and time at which they were determined, fall within the range previously reported by other studies in the ETP [11,41–43,45]. Despite the presence of minor discrepancies in skeletal density across species within sites, these differences did not reach statistical significance when considered at the site, area, and between areas. The only factor for which statistically significant differences in skeletal density were observed for corals of the genus *Pocillopora* was time. The permuted analysis of variance demonstrated a statistically significant reduction in skeletal density in both the Gulf of California entrance (15.4% overall, −0.01512 g CaCO_3_ cm^-3^ per year) and the Oaxaca area (28.6% overall, −0.0227 g CaCO_3_ cm^-3^ per year) over the past two decades. Despite the apparent disparities in the rate of density reduction among different areas (Oaxaca > Gulf of California entrance), analysis of covariance indicates that these differences are not statistically significant. This finding indicates that the observed decrease in skeletal density within *Pocillopora* is not a phenomenon confined to a specific spatial location, but rather a widespread occurrence across a considerable spatial extent. Consequently, we hypothesize that this phenomenon is evident throughout the entire Mexican tropical Pacific region, extending from the Gulf of California entrance to the coast of Oaxaca. However, the extant data on *Pocillopora* density suggest that this pattern is not confined to the Mexican Pacific region but rather occurs on a ETP regional scale [11,41–43,45]. According to the analysis, the skeletal density of *Pocillopora* in the eastern Pacific may be declining at a rate of 0.0217 g CaCO_3_ cm^-3^ per year, which is similar to the carbonate loss suggested for the coast of Oaxaca.

A principal component analysis reveals an increase in sea-surface temperature and a concomitant decline in Ω_ar_ and pH levels in both the Gulf of California entrance and the southern Mexican Pacific over the past two decades. The estimated values of Ω_ar_ and pH estimated are consistent with a basic model that posits that temperature and atmospheric pCO₂ are the predominant influencing factors on the carbonate system in the region. This phenomenon may be attributable to the decadal variability, where other biogeochemical processes (in addition to the CO₂ gas equilibrium) are also active and contributing to this effect [46]. The collective findings suggest a possible correlation between the observed decline in *Pocillopora* skeletal density in the Mexican Pacific and elevated temperatures, as well as decreases in Ω_ar_ and pH in the ocean waters where corals develop. However, a comprehensive record of *Pocillopora* growth parameters (skeletal density, extension rate, calcification rate) remains to be established. In addition, there is a paucity of long-term records of carbon chemistry to validate the modeled data. These facts hinder our capacity to elucidate the mechanism by which skeletons become less dense in response to increased temperature and acidification in both areas. We hypothesize that the cooler waters of the eastern Pacific [47] and the *Pocillopora* species within them demonstrate heightened rates of extension in response to elevated sea-surface temperatures. This phenomenon would result in a reduction in skeletal density, achieved by extending their skeletons to a greater extent with the same or a reduced amount of calcium carbonate. However, *ex situ* evidence suggests that when pocilloporids are acclimated to an elevated temperatures (i.e., 1 °C above their bleaching threshold) for extended periods (six years), skeletons exhibit an increased density but reduced skeletal extension and calcification rates. This phenomenon can be attributed to the prioritization of energy storage over skeletal growth at elevated temperatures [48]. Therefore, regardless of the other variables, environmental conditions (and stress) are the primary drivers of coral calcification. The capacity of coral to undergo changes in metabolism is contingent upon a tenuous energetic equilibrium. This balance is surrogated by energy inputs, which in the ETP are predominantly autotrophic and, to a lesser extent, heterotrophic [49]. Consequently, the constrained energy is apportioned to multiple, ordered processes (e.g., damage repair, somatic growth, calcification, reproduction, energy storage, among others). Imbalances (e.g., OA modifying internal ionic balance) have the potential to influence the organism's processes, including calcification and/or growth. This finding indicates that *Pocillopora* species may prioritize skeletal extension over the thickening of skeletal structures (i.e., increasing the skeletal density) as demonstrated by Carricart-Ganivet and Merino [50] and Carricart-Ganivet [51] for specific Atlantic and Caribbean coral species.

Mollica et al. [5] developed a numerical model of *Porites* skeletal growth. This model establishes a correlation between the skeletal density of corals and the surrounding water in which they develop, attributing this relationship to its impact on the chemistry of coral calcifying fluid. This fluid is located between the coral skeleton and its calicoblastic cell membrane [52]. In contrast to temperature, which has the potential to influence skeletal density or extension depending on the species and the area where corals develop [52–54], Mollica et al. [5] demonstrated that among the variables associated with coral calcification, skeletal density is sensitive to changes in seawater carbonate ion concentration, whereas extension is not. Despite the fact that the model was not generated for *Pocillopora*, the apparent strong biological control over the centers of calcification and the immediately associated fibers during calcification observed in several taxa may be a widespread phenomenon in Scleractinia [5,55–57].

Our local and regional results demonstrate a gradual decline in *Pocillopora* skeletal density over the past two decades, which is consistent with rising temperatures and ocean acidification. This phenomenon aligns with the documented decline in calcification observed in reef-building corals across various regions of the ocean [8]. While data regarding the extent of the affected area are currently unavailable, the coral growth model for the equatorial ETP predicts a gradual reduction in linear extension of *Pocillopora*, estimated at 0.9% per year, due to the effects of increased acidification [11]. The results, when considered collectively, indicate the presence of significant large-scale factors contributing to the reduction in the extent and density of *Pocillopora* spp. skeletons in the ETP, which in turn is leading to a decline in the CaCO_3_ production in the region's reef systems. As Cabral-Tena et al. [18] have demonstrated, the potential for CaCO_3_ production in the ETP reefs is maximized in monogeneric *Pocillopora* reefs. In light of the aforementioned points, two reasonable expectations can be proposed. The initial scenario posits a reduction in the calcification rate of *Pocillopora*, a genus of coral that is estimated to contribute to at least 90% of the total calcification in the region. Prior to the onset of the 2023 heat wave, carbonate balances in eastern Pacific coral ecosystems exhibited a pronounced positive trend in the eastern tropical Pacific (8.22 G) and the coast of Oaxaca (7.21 G) [18,19,58]. Consequently, a reduction in the density of the primary reef builder may have resulted in a 15–29% decline in the net calcification and reef accretion within the coral ecosystems in the eastern tropical Pacific, including the Mexican Pacific. In the absence of a skeletal extension estimate concurrent with the density estimate, an alternative hypothesis is that *Pocillopora* is growing more with an increase in temperature, as has been observed in other species, but the skeleton is less dense [12, and references therein]. Consequently, this phenomenon may render *Pocillopora* reefs more vulnerable to storm damage, wave energy, and bioerosion. Consequently, this phenomenon may potentially result in the flattening of reefs and a reduction in structural complexity observed in eastern Pacific reefs [59]. In summary, the reasonable expectation is that a decrease in calcium carbonate production will result in the possible endangerment of reefs comprising predominantly of *Pocillopora* in the region.

Additionally, the repercussions of ocean acidification on coral reefs, particularly with respect to coral growth, have been the focus of substantial research in recent years. Ocean acidification has been linked to reduced coral cover and diminished growth metrics in multiple coral species [5,60–63], including *Pocillopora* corals in Central American locations [11]. As ocean acidification intensifies, it is likely to emerge as a principal environmental stressor for corals, particularly in locations with pronounced seasonal upwelling (such as the Oaxaca area), which introduces nutrient-rich, low-pH, and low Ω_ar_ waters to the coastline [31,64]. In addition to the remarkable decrease in skeletal density among the primary ETP reef-building species that was observed in this study, the widespread bleaching and substantial mortality that were associated with the 2023 heatwave event have further compounded the situation. According to the data recorded in the Mexican South Pacific, all corals at all sites exhibited complete bleaching during the heatwave, with a mortality rate of 50–93% of the total coral cover [65]. The combination of declining skeletal density, as demonstrated in this study, and extensive coral mortality associated with intensifying and more frequent El Niño events [66] has the potential to disrupt the equilibrium between CaCO₃ accumulation (and the resultant structural complexity of the ecosystem) and losses through microbial, macrobiotic and chemical dissolution. This phenomenon may result in a transition from conditions conducive to elevated CaCO_3_ production to those favoring reduced CaCO_3_ output [67,68]. In cases where environmental pressures are particularly pronounced, a state of negative carbonate budgets, characterized by net reef erosion, could be attained [69,70]. Given that *Pocillopora* is the primary reef-building genus in the ETP region, our findings suggest that the long-term maintenance and reef development in the area may be compromised, potentially leading to reduced reef functionality and altered biodiversity stability, along with associated ecosystem services [19].

The resilience of reefs in the ETP is threatened by a number of factors, including rapid environmental change. Despite the changes in carbonate production and structural complexity, ETP reefs are typically highly porous and consist of uncemented accumulations of CaCO_3_ and poorly cemented sediment matrix [71]. This renders them particularly susceptible to damage from storms and wave action [72]. In the event that all of the aforementioned environmental constraints to reef development in the ETP reach a critical point, the fate of reefs in the region will hinge on the resilience of the coral to adapt to a rapidly changing environment. Notwithstanding the empirical robustness of our analysis and the proposed scenarios, further studies are necessary to address the coupling between biological, chemical, and physical processes. In this regard, it is imperative to develop models that can quantify the coral response to stressors, thereby facilitating the understanding of potential positive feedbacks from calcifiers.

### Potential research gaps and future work

There is a pressing need to identify trends in ocean acidification. This objective can be accomplished by maintaining monitoring efforts oriented towards generating long-term series of the carbonate system in coastal key/representative areas. Moreover, this information will facilitate the identification of the prevailing forcing and controlling factors on sea-surface temperature, pH, and aragonite saturation. This approach will also facilitate the validation of regional and local models, including the one employed in this study.

A notable aspect of this study is the utilization of historical samples for the development of a timeline of coral growth parameters for branching species. However, to obtain long-term databases, it is necessary to encourage the use of current (or custom-made) data repositories. The availability of this information and metadata will facilitate the correction of potential bias resulting from methodological approaches, the definition and adoption of quality criteria, the determination of measurement repetitiveness or precision, and the coupling with other ancillary data.

An evaluation of the effects of density and porosity is imperative. A substantial body of research has demonstrated that ocean acidification leads to an increase in porosity in specific taxa. Consequently, this results in a reduction in skeletal density and an increase in brittleness. However, it is crucial to substantiate this correlation between variables and assess its impact within the context of the ETP, which serves as a natural laboratory for ocean acidification, particularly in light of the diverse reef ecosystems that are dominated by the same *Pocillopora* species.

## Supporting information

S1 Table Number of fragments per morphotype sampled by locality, area, and time. (PDF)

S2 Fig Surface pH (total scale; pHT) and saturation state of aragonite (Ω_ar_) data compilation for both study areas. (PDF)

S3 Fig Modeled pH and saturation state of aragonite (Ω_ar_) for both study areas. (PDF)

S4 Fig Changes in skeletal density across species, times and areas in the Mexican Pacific. Mean (x), median (horizontal line).
(PDF)

S5 Fig Changes in skeletal density across times and areas in the Mexican Pacific. Mean (x), median (horizontal line).
(PDF)

S6 Fig Changes in skeletal density across times in the Mexican Pacific. Mean (x), median (horizontal line).
(PDF)
