# Supplementary material for: Two decades of skeletal density decline in Pocillopora spp. corals in the Mexican Pacific Ocean: Insight into a tropical eastern Pacific acidification scenario?
Source: PLoS One. 2026 Feb 26;21(2):e0342741. doi: 10.1371/journal.pone.0342741 (PMC12944743; doi:10.1371/journal.pone.0342741)
Supplement: S1 Table — (PDF) [file pone.0342741.s001.pdf]

Region	Year	Locality	Species	Skeletal Density
La Paz	1996	B. Chileno	Pdam	2.27
La Paz	1996	B. Chileno	Pver	2.08
La Paz	1996	Cabo Pulmo	Pdam	2.47
La Paz	1996	Cabo Pulmo	Pver	1.78
La Paz	1996	Cabo Pulmo	Pver	2.42
La Paz	1996	Isla Espíritu Santo	Pdam	2.11
La Paz	1996	Isla San Jose	Pdam	1.97
La Paz	1996	Isla San Jose	Pdam	1.99
La Paz	1996	Pichilingue	Pdam	1.99
La Paz	1996	Punta Arenas	Pcap	2.51
La Paz	1996	Punta Arenas	Pcap	2.34
La Paz	1996	Punta Arenas	Pdam	2.49
La Paz	1996	Punta Arenas	Pmea	2.55
La Paz	1996	Punta Arenas	Pmea	2.07
La Paz	1996	Punta Arenas	Pmea	1.82
La Paz	1996	Punta Arenas	Pver	2.26
La Paz	1996	Punta Arenas	Pver	2.46
La Paz	1996	Punta Perico	Pcap	2.29
La Paz	1996	Punta Perico	Pcap	2.46
La Paz	1996	Punta Perico	Pdam	2.26
La Paz	1996	Punta Perico	Pmea	2.13
La Paz	1996	Punta Perico	Pmea	2.24
La Paz	1996	Punta Perico	Pver	2.30
La Paz	1996	Punta Perico	Pver	2.03
La Paz	1996	Punta Perico	Pver	2.26
La Paz	1996	Punta Perico	Pver	1.72
La Paz	1996	Punta Perico	Pver	2.30
La Paz	1996	Punta Perico	Pver	2.32
La Paz	1996	Punta Perico	Pver	2.35
La Paz	1996	Punta Perico	Pver	2.39
La Paz	1996	Punta Perico	Pver	2.07
La Paz	2016	Bonanza	Pdam	1.62
La Paz	2016	Bonanza	Pver	2.30
La Paz	2016	Bonanza	Pver	1.63
La Paz	2016	Bonanza	Pver	1.94
La Paz	2016	Calerita	Pdam	2.09
La Paz	2016	Calerita	Pele	1.94
La Paz	2016	Calerita	Pver	1.88
La Paz	2016	Punta Arenas	Pgra	2.06
La Paz	2016	Punta Arenas	Pgra	1.94
La Paz	2016	Punta Arenas	Pgra	1.82
La Paz	2016	Punta Arenas	Pver	2.30

La Paz	2016	Punta Arenas	Pver	2.36
La Paz	2016	Punta Arenas	Pver	2.21
La Paz	2016	Punta Arenas	Pver	2.31
La Paz	2016	Punta Arenas	Pver	2.16
La Paz	2016	Punta Arenas	Pver	2.32
La Paz	2016	San Gabriel	Pdam	1.78
La Paz	2016	San Gabriel	Pdam	1.29
La Paz	2016	San Gabriel	Pdam	2.35
La Paz	2016	San Gabriel	Pdam	1.97
La Paz	2016	San Gabriel	Pdam	2.32
La Paz	2016	San Gabriel	Pdam	1.52
La Paz	2016	San Gabriel	Pdam	2.10
La Paz	2016	Swany Reef	Pcap	1.67
La Paz	2016	Swany Reef	Pcap	2.09
La Paz	2016	Swany Reef	Pcap	1.30
La Paz	2016	Swany Reef	Pcap	1.19
La Paz	2016	Swany Reef	Pcap	2.02
La Paz	2016	Swany Reef	Pcap	1.64
La Paz	2016	Swany Reef	Pver	1.31
Oax	1994	Dársena	Pdam	2.04
Oax	1994	Dársena	Pdam	1.90
Oax	1994	Dársena	Pver	1.76
Oax	1994	Isla Montosa	Pgra	1.31
Oax	1994	Isla Montosa	Pgra	1.67
Oax	1994	Isla Montosa	Pgra	1.38
Oax	1994	Isla Montosa	Pgra	1.84
Oax	1994	Isla Montosa	Pgra	2.10
Oax	1994	Isla Montosa	Pgra	1.82
Oax	1994	Isla Montosa	Pgra	2.04
Oax	1994	Isla Montosa	Pgra	2.22
Oax	1994	Isla Montosa	Pgra	1.79
Oax	1994	Isla Montosa	Pgra	2.06
Oax	1994	Isla Montosa	Pgra	1.88
Oax	1994	Isla Montosa	Pmea	1.67
Oax	1994	Isla Montosa	Pmea	1.69
Oax	1994	La Entrega	Pdam	2.07
Oax	1994	La Entrega	Pdam	2.03
Oax	1994	La Entrega	Pdam	1.71
Oax	1994	La Entrega	Pver	1.09
Oax	1994	La Guerrilla	Pcap	1.66
Oax	1994	La India	Pver	1.90
Oax	1994	La Prima	Pcap	1.72
Oax	1994	La Prima	Pcap	2.10

Oax	1994	La Prima	Pgra	2.00
Oax	1994	La Prima	Pgra	1.65
Oax	1994	La Prima	Pgra	1.70
Oax	1994	La Prima	Pgra	1.93
Oax	1994	La Prima	Pgra	1.71
Oax	1994	La Tijera	Pver	2.00
Oax	1994	La Tijera	Pver	1.23
Oax	1994	Mazunte	Pdam	2.26
Oax	1994	Mazunte	Pdam	1.50
Oax	1994	Mazunte	Pver	2.14
Oax	1994	Riscalillo	Pcap	1.93
Oax	1994	Riscalillo	Pgra	2.07
Oax	1994	Salchi	Pdam	1.80
Oax	1994	Salchi	Pver	1.76
Oax	2017	Dársena	Pdam	2.01
Oax	2017	Dársena	Pdam	1.03
Oax	2017	Dársena	Pver	1.02
Oax	2017	Dársena	Pver	1.26
Oax	2017	Dársena	Pver	1.24
Oax	2017	Dársena	Pver	1.21
Oax	2017	Estacahuite	Pgra	1.23
Oax	2017	Estacahuite	Pgra	1.24
Oax	2017	Estacahuite	Pmea	1.65
Oax	2017	Estacahuite	Pmea	1.69
Oax	2017	Estacahuite	Pver	1.25
Oax	2017	Estacahuite	Pver	1.85
Oax	2017	Isla Montosa	Pver	1.38
Oax	2017	Isla Montosa	Pver	1.34
Oax	2017	Isla Montosa	Pver	1.42
Oax	2017	Isla Montosa	Pver	1.71
Oax	2017	Isla Montosa	Pver	1.69
Oax	2017	Isla Montosa	Pver	1.88
Oax	2017	Isla Montosa	Pver	1.69
Oax	2017	La Entrega	Pdam	1.29
Oax	2017	La Entrega	Pdam	0.77
Oax	2017	La Entrega	Pdam	1.23
Oax	2017	La Entrega	Pdam	0.81
Oax	2017	La Entrega	Pdam	1.11
Oax	2017	La Entrega	Pdam	1.66
Oax	2017	Maguey	Pgra	1.40
Oax	2017	Maguey	Pgra	1.26
Oax	2017	Maguey	Pver	0.92
Oax	2017	Maguey	Pver	0.88

Oax	2017	Maguey	Pver	1.40
Oax	2017	Maguey	Pver	0.87
Oax	2017	Maguey	Pver	0.89
Oax	2017	Panteón	Pdam	1.49
Oax	2017	Panteón	Pdam	1.24
Oax	2017	Panteón	Pele	1.13
Oax	2017	Panteón	Pver	1.04
Oax	2017	Panteón	Pver	1.14
Oax	2017	Panteón	Pver	0.91
