# Supplementary material for: Two decades of skeletal density decline in Pocillopora spp. corals in the Mexican Pacific Ocean: Insight into a tropical eastern Pacific acidification scenario?
Source: PLoS One. 2026 Feb 26;21(2):e0342741. doi: 10.1371/journal.pone.0342741 (PMC12944743; doi:10.1371/journal.pone.0342741)
Supplement: S2 Fig — (PDF) [file pone.0342741.s002.pdf]

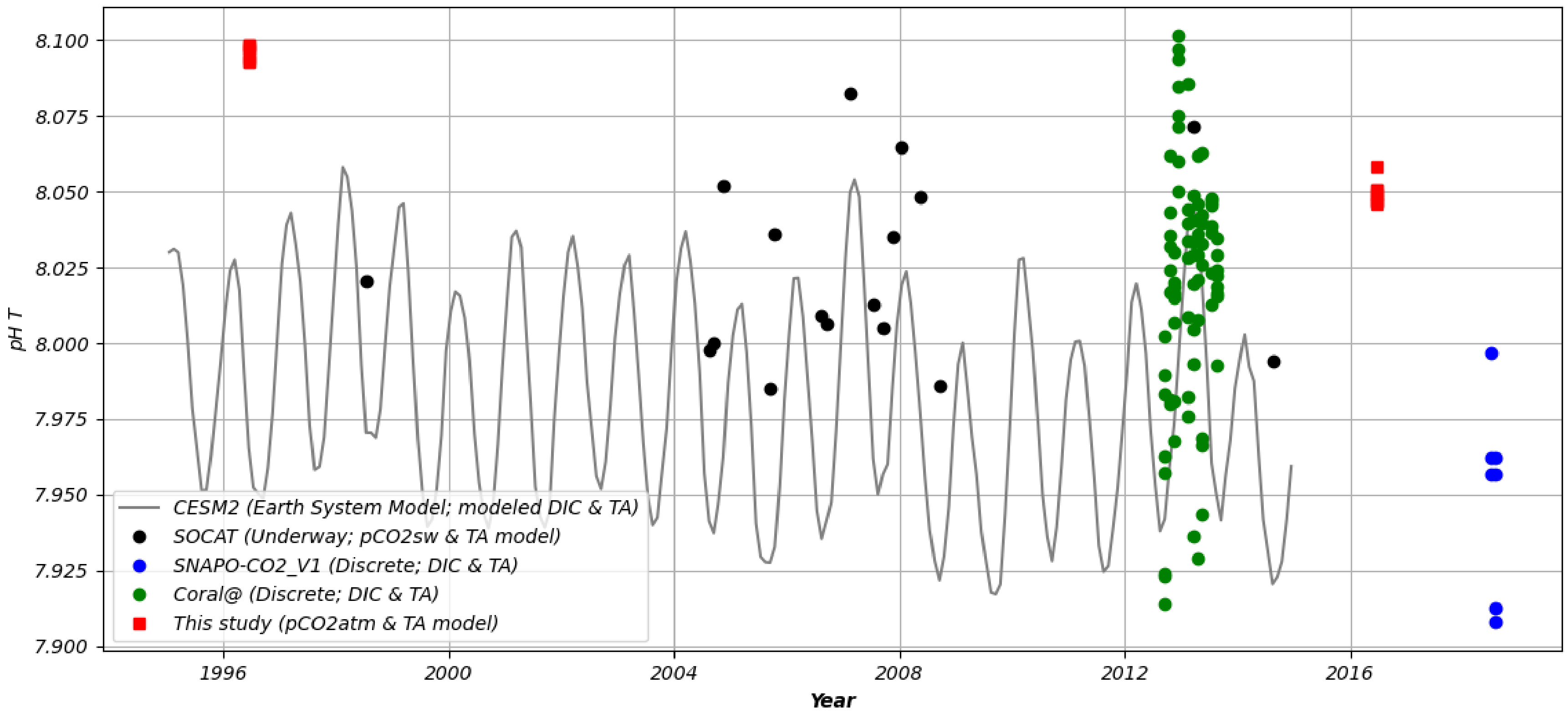

Fig. A. pHT time series for the region near to La Paz. Data was obtained from: (1) global models (CESM2; https://www.cesm.ucar.edu/models/cesm2), (2) surface ocean CO_2 atlas (SOCAT; https://socat.info/), (3) global databases (SNAPO-CO2-v1 dataset; Metzl et al. 2025), (4) local databases (Coral@; Norzagaray et al. 2017), and (4) data using pCO2atm and modeled TA. Discrete data show an ample variability towards lower values, mainly related to the influence of subsurface water (cold and carbon-rich water) and/or the presence of water masses with a distinct biogeochemical condition on a seasonal scale (mostly during winter). Underway and modeled data (global and simple models), both surface data, fall within a similar range. Additionally, the CESM2 data, although limited in amplitude, reflects average conditions in the region, and show a slight negative trend for pHT.

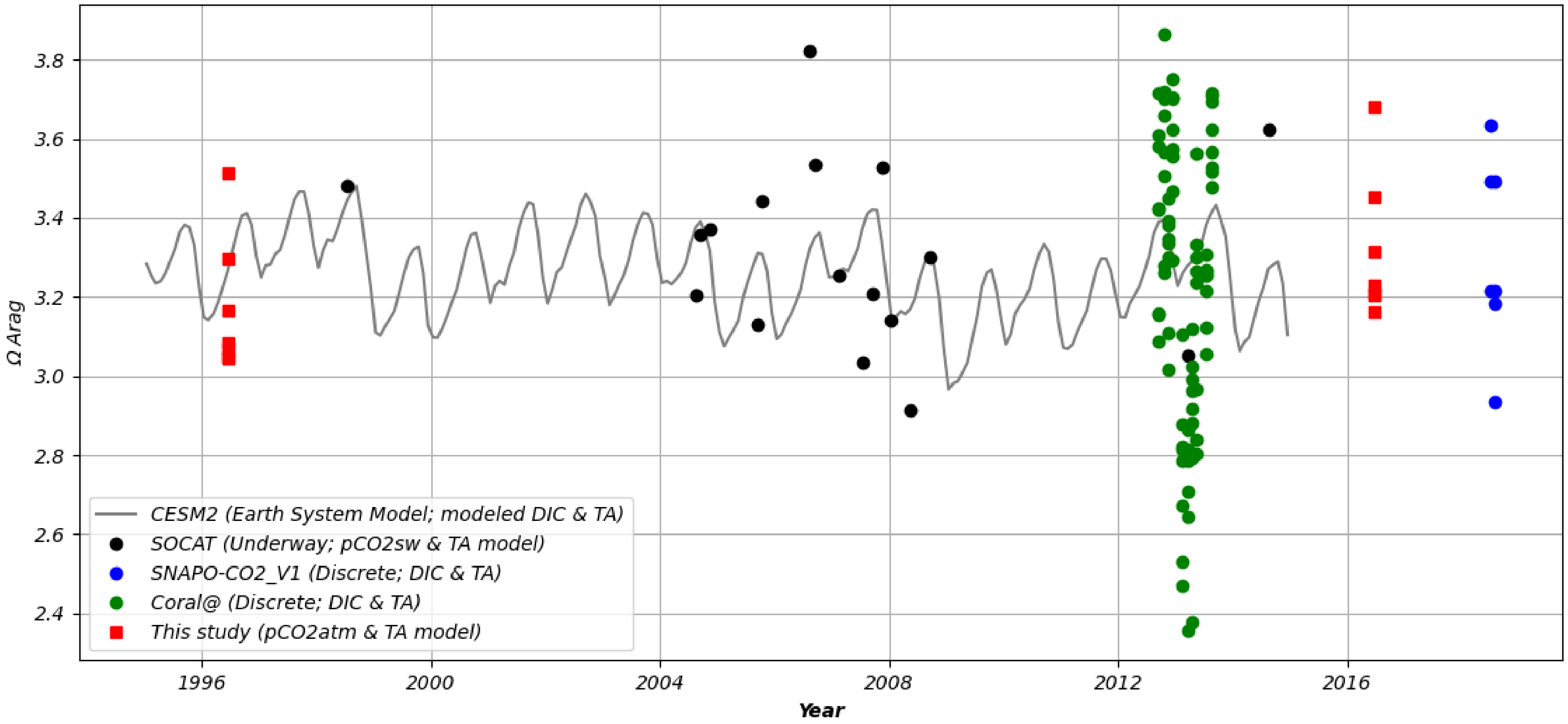

Fig. B. Ω_{ar} time series for the region near to La Paz. Data was obtained from: (1) global models (CESM2; https://www.cesm.ucar.edu/models/cesm2), (2) surface ocean CO_2 atlas (SOCAT; https://socat.info/), (3) global databases (SNAPO-CO2-v1 dataset; Metzl et al. 2025), (4) local databases (Coral@; Norzagaray et al. 2017), and (5) data using pCO2atm and modeled TA. Similar to pHT (Fig. A), discrete data show a wide variability, and CESM2 data indicate a negative trend.

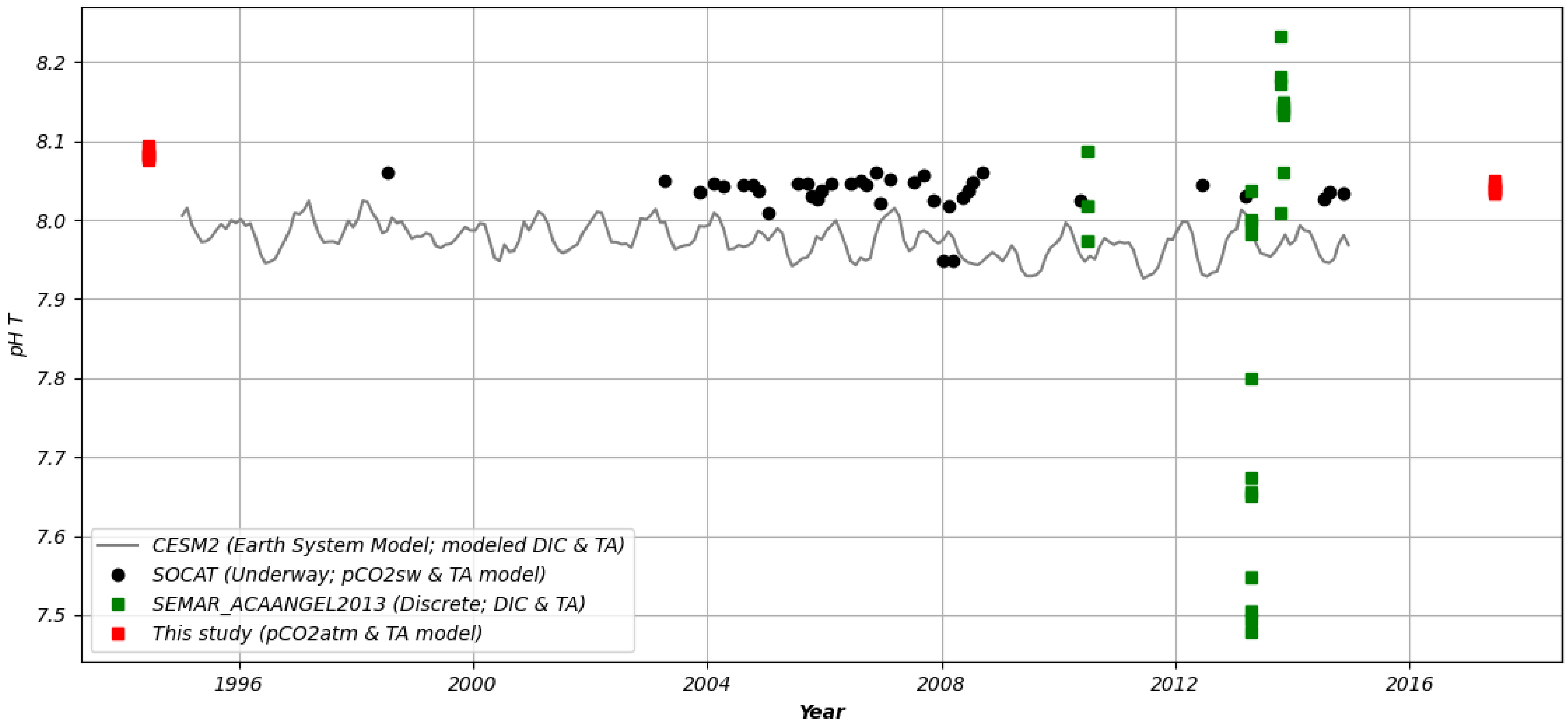

Fig. C. pHT time series for the region near the coast of Oaxaca. Data was obtained from: (1) global models (CESM2; https://www.cesm.ucar.edu/models/cesm2), (2) surface ocean CO_2 atlas (SOCAT; https://socat.info/), (3) local databases (SEMAR_ACAANGEL2013; Chapa-Balcorta et al. 2015), and (4) data using pCO2atm and modeled TA. Again, discrete data showed a strong seasonal variability, associated with intense upwelling events (named 'Tehuanos'). Underway and surface modeled data fall within a similar range. The CESM2 data show a negative trend, similar to La Paz.

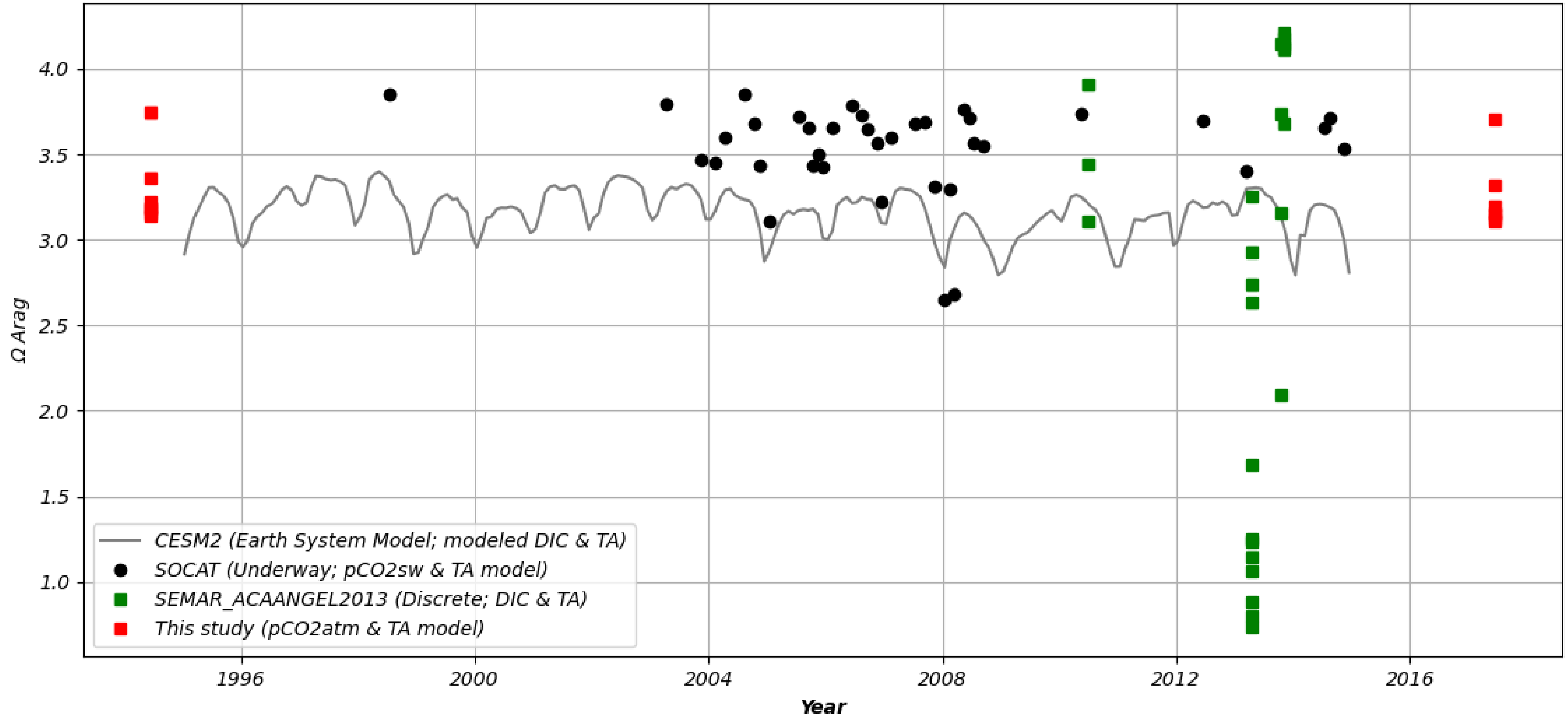

Fig. D. Ω_{ar} time series for the region near the coast of Oaxaca. Data was obtained from: (1) global models (CESM2; https://www.cesm.ucar.edu/models/cesm2), (2) surface ocean CO_2 atlas (SOCAT; https://socat.info/), (3) local databases (SEMAR_ACAANGEL2013; Chapa-Balcorta et al. 2015), and (4) data using pCO2atm and modeled TA. Similar to pHT, discrete data show marked seasonal variability, associated with intense upwelling events, during which Ω_{ar} values can be near to subsaturation. In contrast, underway and surface modeled data fall within a considerably narrower range (3 to 4 units). Again, CESM2 data shows a similar slight negative trend.
