# Supplementary material for: Two decades of skeletal density decline in Pocillopora spp. corals in the Mexican Pacific Ocean: Insight into a tropical eastern Pacific acidification scenario?
Source: PLoS One. 2026 Feb 26;21(2):e0342741. doi: 10.1371/journal.pone.0342741 (PMC12944743; doi:10.1371/journal.pone.0342741)
Supplement: S3 Fig — (PDF) [file pone.0342741.s003.pdf]

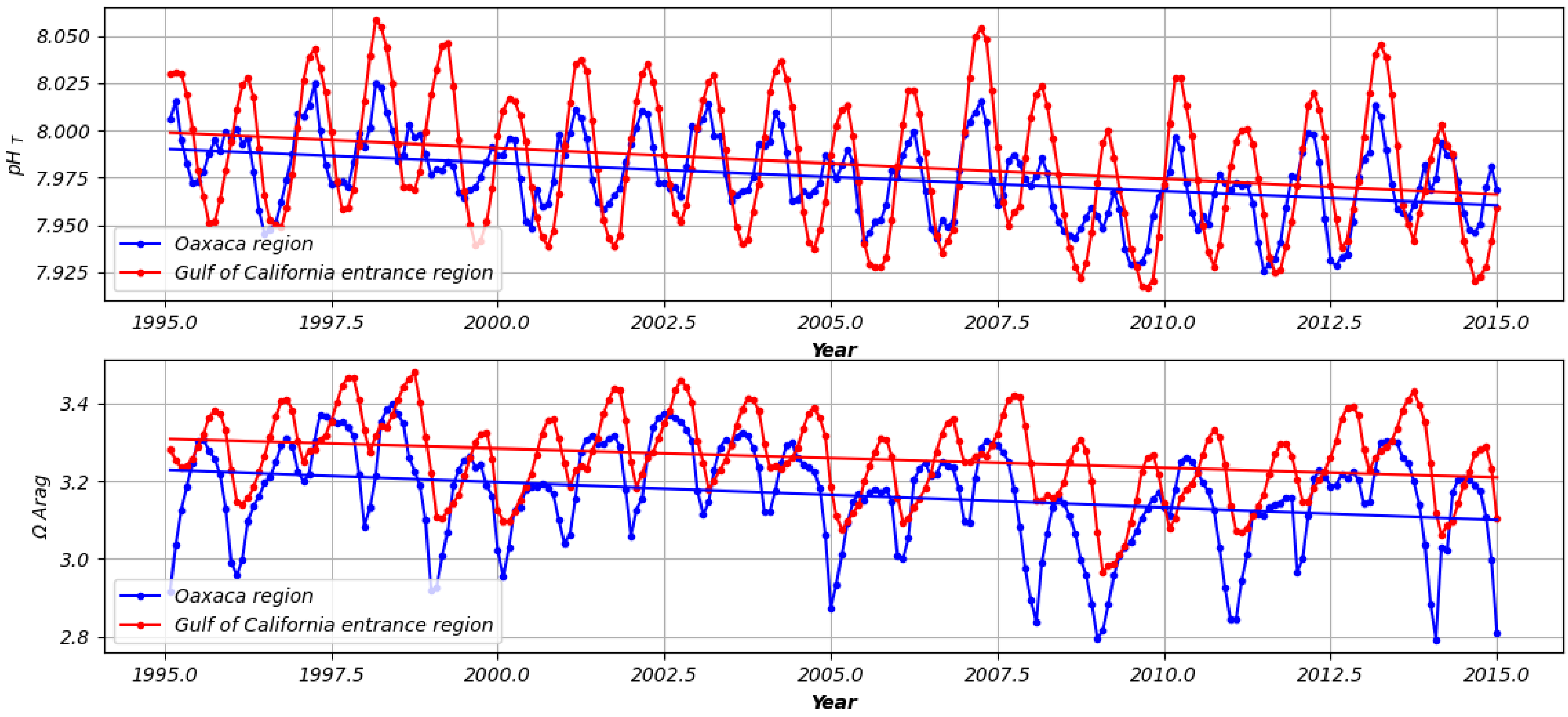

Figure. Monthly time series of surface pHT (total scale; above) and ΩArag (below) obtained from CESM2 model (ttps://www.cesm.ucar.edu/models/cesm2) for La Paz (red dotted line) and Oaxaca (blue dotted line) regions. Simple linear regression lines are shown (solid lines) for both CO2 system parameters. pHT shows a significant negative trend for La Paz ($y = -0.001x + 11.28$; $F_{1,238} = 18.4$; $p<0.05$) and Oaxaca ($y = -0.001x + 10.96$; $F_{1,238} = 47.2$; $p<0.05$). Similarly, Ω_{ar} displays a significant negative trend in La Paz ($y = -0.005x + 13.22$; $F_{1,238} = 18.4$; $p<0.05$) and Oaxaca ($y = -0.006x + 16.17$; $F_{1,238} = 21.4$; $p<0.05$). These negative trends are consistent with a progressive ocean acidification related to a continuous pCO2atm forcing, which has been reported. However, despite the similar trends, is worth to notice that model outputs are limited in spatial and temporal resolution, which may smooth local variability and mask oceanic process at regional scale (such as intense upwelling events).
