# Supplementary figures and images for: Two decades of skeletal density decline in Pocillopora spp. corals in the Mexican Pacific Ocean: Insight into a tropical eastern Pacific acidification scenario?
Source: PLoS One. 2026 Feb 26;21(2):e0342741. doi: 10.1371/journal.pone.0342741 (PMC12944743; doi:10.1371/journal.pone.0342741)

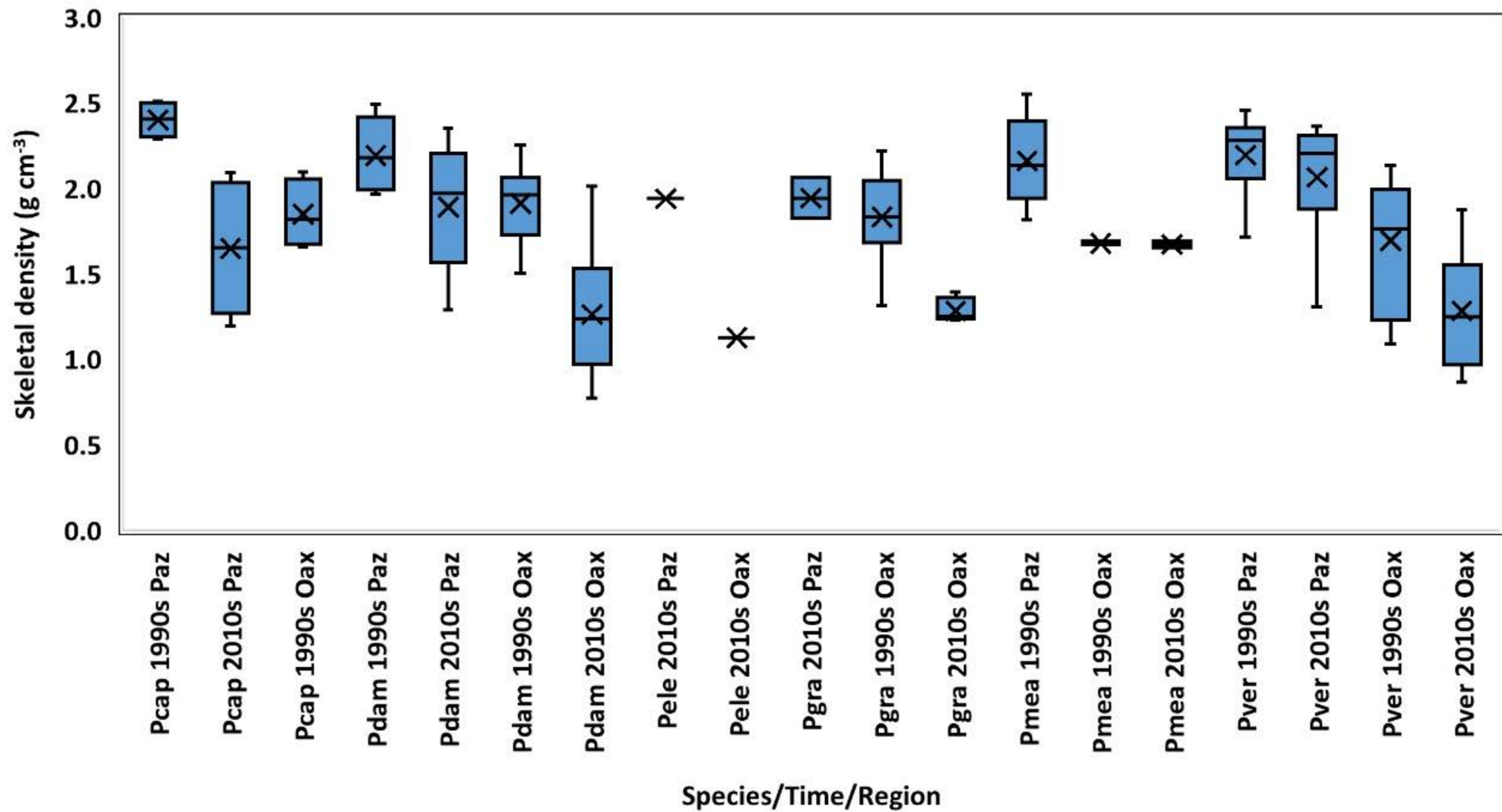

Supplement: S4 Fig — Mean (x), median (horizontal line). (PDF) [file pone.0342741.s004.pdf]

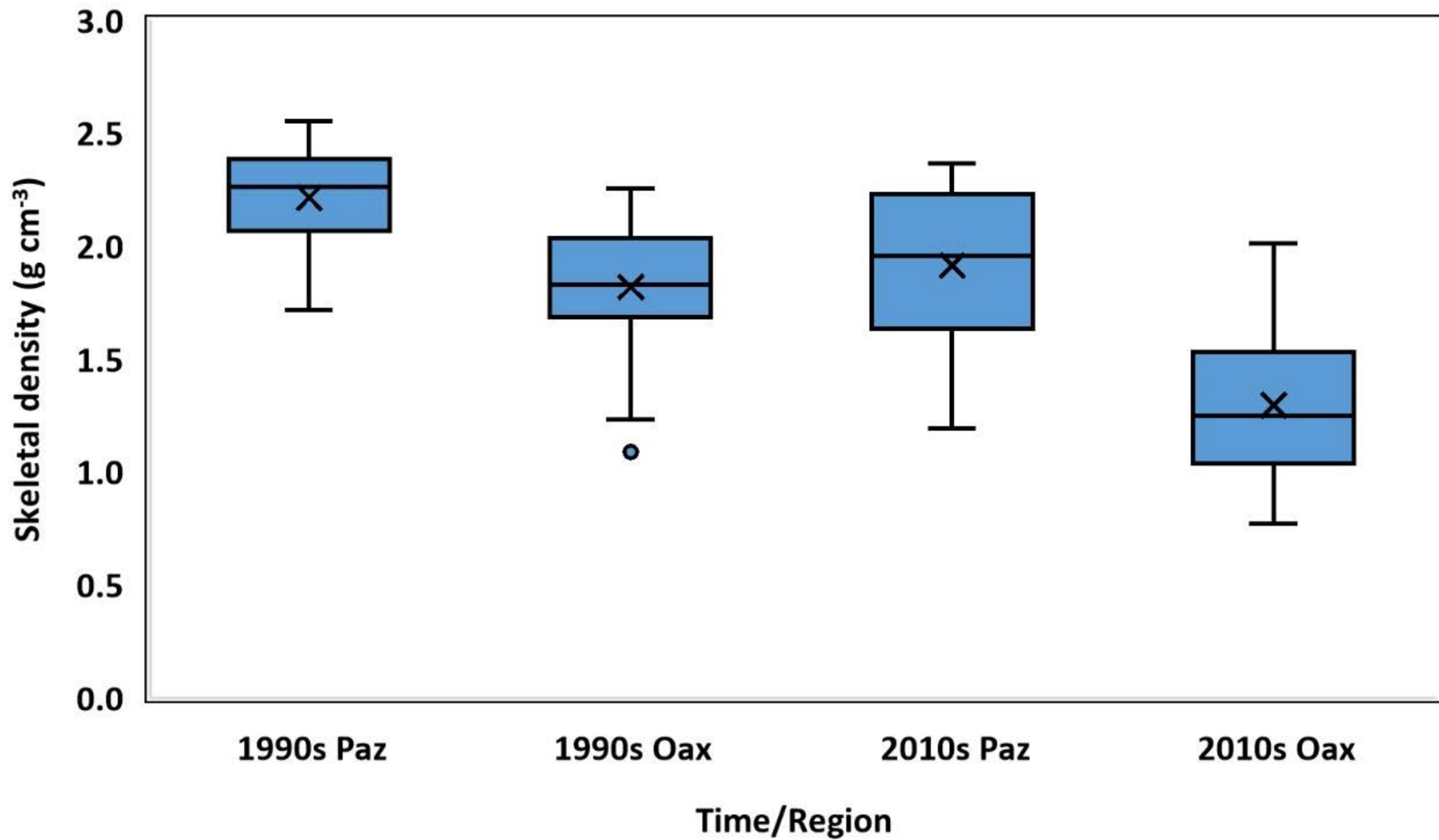

Supplement: S5 Fig — Mean (x), median (horizontal line). (PDF) [file pone.0342741.s005.pdf]

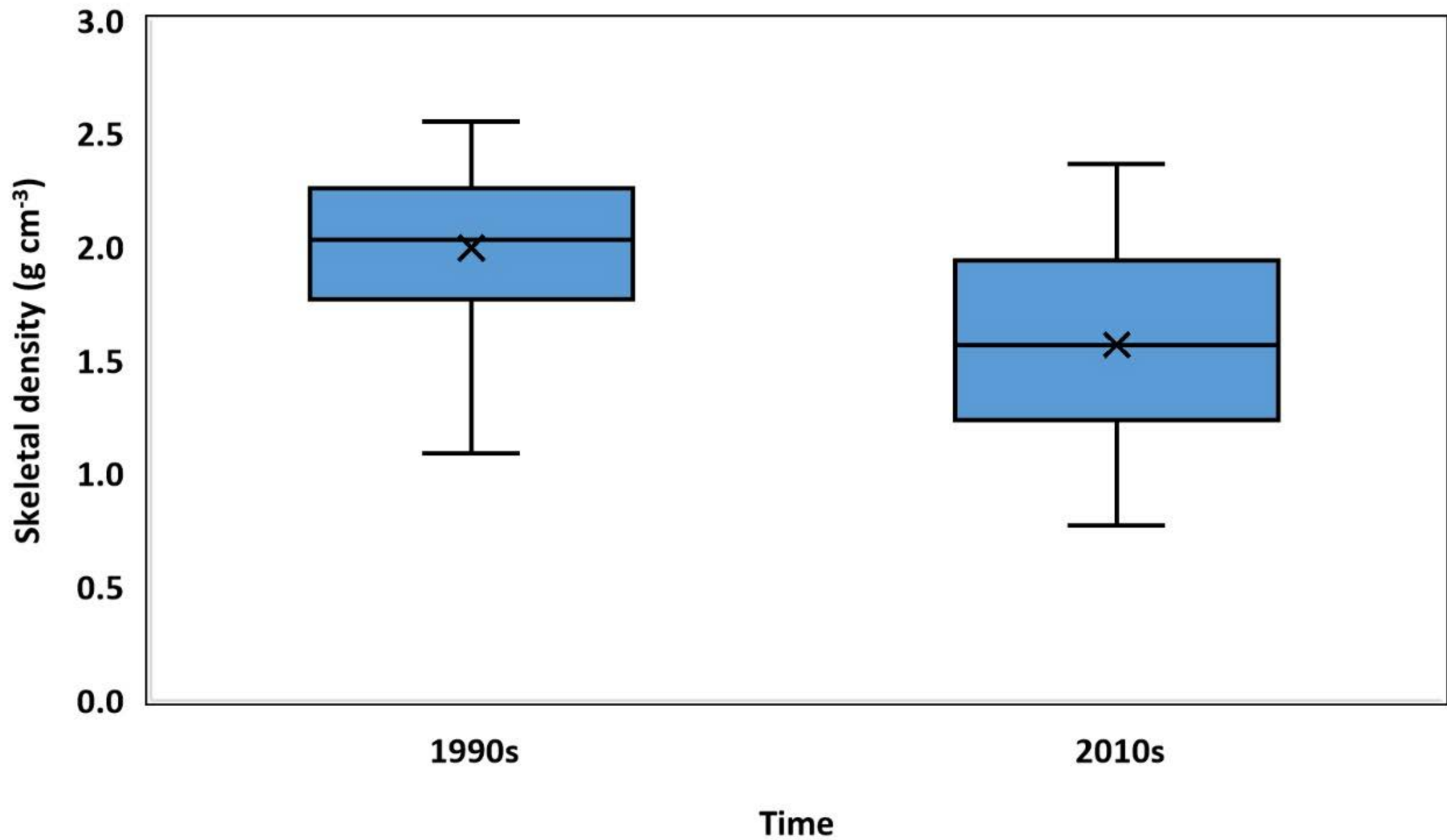

Supplement: S6 Fig — Mean (x), median (horizontal line). (PDF) [file pone.0342741.s006.pdf]
